# Empirical Validation of a Hypothesis of the Hormetic Selective Forces Driving the Evolution of Longevity Regulation Mechanisms

**DOI:** 10.3389/fgene.2016.00216

**Published:** 2016-12-06

**Authors:** Alejandra Gomez-Perez, Pavlo Kyryakov, Michelle T. Burstein, Nimara Asbah, Forough Noohi, Tania Iouk, Vladimir I. Titorenko

**Affiliations:** Department of Biology, Concordia UniversityMontreal, QC, Canada

**Keywords:** yeast, aging, longevity, natural aging-delaying compounds, ecosystems, evolution, longevity regulation mechanisms

## Abstract

Exogenously added lithocholic bile acid and some other bile acids slow down yeast chronological aging by eliciting a hormetic stress response and altering mitochondrial functionality. Unlike animals, yeast cells do not synthesize bile acids. We therefore hypothesized that bile acids released into an ecosystem by animals may act as interspecies chemical signals that generate selective pressure for the evolution of longevity regulation mechanisms in yeast within this ecosystem. To empirically verify our hypothesis, in this study we carried out a three-step process for the selection of long-lived yeast species by a long-term exposure to exogenous lithocholic bile acid. Such experimental evolution yielded 20 long-lived mutants, three of which were capable of sustaining their considerably prolonged chronological lifespans after numerous passages in medium without lithocholic acid. The extended longevity of each of the three long-lived yeast species was a dominant polygenic trait caused by mutations in more than two nuclear genes. Each of the three mutants displayed considerable alterations to the age-related chronology of mitochondrial respiration and showed enhanced resistance to chronic oxidative, thermal, and osmotic stresses. Our findings empirically validate the hypothesis suggesting that hormetic selective forces can drive the evolution of longevity regulation mechanisms within an ecosystem.

## Introduction

We have identified lithocholic acid (LCA), a bile acid, as a potent geroprotector that delays chronological aging in the yeast *Saccharomyces cerevisiae* (Goldberg et al., 2010b). Several other bile acids can decelerate yeast chronological aging as well, although to a lesser extent than LCA (Goldberg et al., 2010b). We demonstrated that exogenously added LCA enters the yeast cell, accumulates in both mitochondrial membranes, causes a remodeling of mitochondrial membrane lipidome, alters morphology and functionality of mitochondria, and allows mitochondria to establish and sustain an aging-delaying pattern of the entire cell (Burstein et al., 2012b; Beach et al., 2013, 2015b; Burstein and Titorenko, 2014; Medkour and Titorenko, 2016).

A body of evidence indicates that endogenous and exogenously added bile acids can extend healthy lifespan in laboratory mammals and nematodes (Amador-Noguez et al., 2004, 2007; Motola et al., 2006; Gems, 2007; Gerisch et al., 2007; Russell and Kahn, 2007; Gems and Partridge, 2008; Ramalho et al., 2008; Thomas et al., 2008; Amaral et al., 2009; Hylemon et al., 2009; Lefebvre et al., 2009; Monte et al., 2009; Tiwari and Maiti, 2009; Vallim and Edwards, 2009; Goldberg et al., 2011, 2013; Pols et al., 2011; Wollam et al., 2011, 2012; Lee and Schroeder, 2012; Chiang, 2013; de Aguiar Vallim et al., 2013; Groen and Kuipers, 2013; Li and Chiang, 2013, 2014; Magner et al., 2013; Arlia-Ciommo et al., 2014b; Mahanti et al., 2014). Bile acids are beneficial to health and longevity of animals not only because they accelerate the emulsification and absorption of dietary lipids and fat-soluble vitamins, affect the composition and proliferation of the intestinal microbial flora, and support the maintenance of organismal sterol homeostasis (Thomas et al., 2008; Amaral et al., 2009; Hylemon et al., 2009; Lefebvre et al., 2009; Monte et al., 2009; de Aguiar Vallim et al., 2013). Bile acids extend healthy lifespan in animals also because they act as signaling molecules that enable to sustain lipid, glucose, and energy homeostasis (Motola et al., 2006; Gerisch et al., 2007; Russell and Kahn, 2007; Ramalho et al., 2008; Thomas et al., 2008; Amaral et al., 2009; Hylemon et al., 2009; Lefebvre et al., 2009; Monte et al., 2009; Tiwari and Maiti, 2009; Vallim and Edwards, 2009; Goldberg et al., 2011, 2013; Pols et al., 2011; Wollam et al., 2011, 2012; Lee and Schroeder, 2012; Chiang, 2013; de Aguiar Vallim et al., 2013; Groen and Kuipers, 2013; Li and Chiang, 2013; Magner et al., 2013; Arlia-Ciommo et al., 2014b; Mahanti et al., 2014). Moreover, bile acids extend healthy lifespan in animals because these mildly toxic molecules with detergent-like properties can activate detoxification of xenobiotics, thus promoting chemical hormesis and operating as endobiotic regulators of aging that improve health and prolong longevity (Amador-Noguez et al., 2004, 2007; Gems, 2007; Russell and Kahn, 2007; Gems and Partridge, 2008; Burstein et al., 2012a; Arlia-Ciommo et al., 2014b; Li and Chiang, 2014; Medkour and Titorenko, 2016).

Although LCA and other bile acids delay yeast chronological aging, yeast cells do not synthesize these molecules (Lefebvre et al., 2009; Monte et al., 2009; Goldberg et al., 2010b). We therefore hypothesized that bile acids released into the environment by animals composing an ecosystem may act as interspecies chemical signals that create selective pressure for the evolution of longevity regulation mechanisms in yeast within this ecosystem (Goldberg et al., 2010a; Burstein et al., 2012a). Our hypothesis posits the following: (1) only yeast exposed to exogenous bile acids can develop mechanisms of protection against cellular damage caused by these external stress agents and hormetic stimuli; (2) some of these mechanisms developed against bile acid-induced cellular damage can also protect yeast against damage and stress accumulated purely with age; (3) only those yeast species that have developed (due to exposure to exogenous bile acids) the most protective mechanisms against bile acid-induced cellular damage can also develop protective mechanisms against damage and stress accumulated with age; and (4) these yeast species are therefore expected to live longer (Goldberg et al., 2010a,b; Burstein et al., 2012a). In this hypothesis, the presence of exogenous bile acids creates hormetic selective force that drives the evolution of not only protective mechanisms against bile acid-induced cellular damage but also longevity regulation mechanisms that protect against damage and stress accumulated with age. Moreover, this hypothesis suggests that yeast cells that are not exposed to exogenous bile acids cannot develop mechanisms of protection against cellular damage caused by these mildly toxic molecules (Goldberg et al., 2010a,b; Burstein et al., 2012a). Thus, these yeast cells are unable to develop mechanisms of protection against damage and stress accumulated purely with age.

To empirically verify our hypothesis, in this study we conducted a multistep selection of long-lived yeast mutants by a lasting exposure to LCA under laboratory conditions. We selected 20 of such mutants. Three of these mutants were able to maintain their greatly extended lifespans following numerous passages in medium without LCA. We provide evidence that the extended longevity of each of the three long-lived yeast mutants is a dominant polygenic trait caused by mutations in more than two nuclear genes. We demonstrate that each of these mutants exhibits significant changes to the age-related dynamics of mitochondrial respiration and displays enhanced resistance to chronic oxidative, thermal, and osmotic stresses.

## Materials and Methods

### Yeast Strains, Media, and Growth Conditions

The wild-type (WT) strain *S. cerevisiae* BY4742 (*MATα his3Δ leu2Δ0 lys2Δ ura3Δ0*) from Thermo Scientific/Open Biosystems and long-lived mutant species 3, 5, and 12 derived from this strain were grown in liquid YP medium (1% yeast extract, 2% peptone) initially containing 0.2% glucose as a carbon source, with or without LCA, as detailed in Section “Results.” Cells were cultured at 30°C with rotational shaking at 200 rpm in Erlenmeyer flasks at a “flask volume/medium volume” ratio of 5:1.

### Chronological Lifespan Assay

A sample of cells was taken from a culture at a certain day following cell inoculation into the medium. A fraction of the sample was diluted in order to determine the total number of cells using a hemocytometer. Another fraction of the cell sample was diluted and serial dilutions of cells were plated in duplicate onto YEP [1% (w/v) yeast extract, 2% (w/v) peptone] plates containing 2% (w/v) glucose as carbon source. After 2 days of incubation at 30°C, the number of colony forming units (CFU) per plate was counted. The number of CFU was defined as the number of viable cells in a sample. For each culture, the percentage of viable cells was calculated as follows: (number of viable cells per ml/total number of cells per ml) × 100. The percentage of viable cells in mid-logarithmic growth phase was set at 100%.

### Mating Assay

A small patch of cells of mating type α [i.e., the haploid WT strain BY4741 (*MATa his3Δ1 leu2Δ0 met15Δ0 ura3Δ0*)] was applied to the surface of a master YEPD (1% yeast extract, 2% peptone, 2% glucose, 2% agar) plate. 10^6^ cells of mating type α [i.e., the haploid WT strain BY4742 (*MATα his3Δ1 leu2Δ0 lys2Δ0 ura3Δ0*) or the selected long-lived haploid mutant strains 3, 5, or 12] were spread on the surface of a separate crossing plate with YEPD medium. The master plate was replica plated onto a lawn of cells on each of the four crossing plates; different velvet was used for each crossing plate. The crossing plates were incubated overnight at 30°C. Each of the four crossing plates was then replica plated onto a synthetic minimal YNB medium plate (0.67% yeast nitrogen base without amino acids, 2% glucose, 2% agar) supplemented with 20 mg/l L-histidine, 30 mg/l L-leucine and 20 mg/l uracil. These plates were incubated overnight at 30°C. A positive mating reaction between cells of the haploid WT strain BY4741 (*MATa his3Δ1 leu2Δ0 met15Δ0 ura3Δ0*) and cells of the haploid WT strain BY4742 (*MATα his3Δ1 leu2Δ0 lys2Δ0 ura3Δ0*) or cells of each of the selected long-lived haploid mutant strains 3, 5, or 12 resulted in confluent growth of diploid cells on a YNB plate (supplemented with L-histidine, L-leucine, and uracil) at the position of a patch of haploid BY4741 cells.

### Sporulation Assay

Cells of the diploid strains recovered in the mating assay were spread on a separate SPO (0.1% yeast extract, 1% potassium acetate, 0.05% glucose, 2% agar) plate supplemented with 20 mg/l L-histidine, 30 mg/l L-leucine, and 20 mg/l uracil. The plates were incubated at 30°C for 5–6 days, until numerous asci were observed microscopically.

### Dissection of Asci, Separation of Ascospores, and Tetrad Analysis

An inoculating loop was used to resuspend spores from the lawn on SPO plates in 50 μl of 0.2 mg/ml Zymolyase 20T solution in sterile water. The suspension of spores was incubated for 10 min at room temperature. A total of 450 μl of sterile water was gently added to the suspension of spores. The mix was then incubated for 30–40 min at room temperature. An inoculating loop was used to spread an aliquot of digested asci onto a tetrad dissecting YEPDD (1% yeast extract, 2% peptone, 2% glucose, 4% agar) plate. Dissection of asci, separation of ascospores, and tetrad analysis were performed according to established procedures (Sherman, 2002; Amberg et al., 2005; Boone et al., 2016).

### Oxygen Consumption Assay

A sample of cells was taken from a culture at a certain time-point. Cells were pelleted by centrifugation and resuspended in 1 ml of fresh YP medium containing 0.2% glucose. Oxygen uptake by cells was measured continuously in a 2-ml stirred chamber using a custom-designed biological oxygen monitor (Science Technical Center of Concordia University) equipped with a Clark-type oxygen electrode.

### Plating Assays for the Analysis of Resistance to Various Chronic Stresses

For the analysis of hydrogen peroxide (oxidative stress) resistance, serial dilutions (1:10 to 1:10^5^) of WT and mutant cells removed from each culture at various time-points were spotted onto two sets of plates. One set of plates contained YP medium with 2% glucose alone, whereas the other set contained YP medium with 2% glucose supplemented with 5 mM hydrogen peroxide. Pictures were taken after a 3-day incubation at 30°C.

For the analysis of thermal stress resistance, serial dilutions (1:10 to 1:10^5^) of WT and mutant cells removed from each culture at various time-points were spotted onto two sets of plates containing YP medium with 2% glucose. One set of plates was incubated at 30°C. The other set of plates was initially incubated at 55°C for 30 min, and was then transferred to 30°C. Pictures were taken after a 3-day incubation at 30°C.

For the analysis of osmotic stress resistance, serial dilutions (1:10 to 1:10^5^) of WT and mutant cells removed from each culture at various time-points were spotted onto two sets of plates. One set of plates contained YP medium with 2% glucose alone, whereas the other set contained YP medium with 2% glucose supplemented with 1 M sorbitol (S6021; Sigma). Pictures were taken after a 3-day incubation at 30°C.

### Statistical Analysis

Statistical analysis was performed using Microsoft Excel’s (2010) Analysis ToolPak-VBA. All data on cell survival are presented as mean ± SEM. The *p* values for comparing the means of two groups using an unpaired two-tailed *t*-test were calculated with the help of the GraphPad Prism 7 statistics software. The log-rank test for comparing each pair of survival curves was performed with GraphPad Prism 7. Two survival curves were considered statistically different [and two strains were concluded to exhibit a significant difference in chronological lifespan (CLS)] if the *p* value was less than 0.05.

## Results

### The LCA-Driven Selection of Long-Lived Yeast Species under Laboratory Conditions

To empirically verify our hypothesis suggesting that hormetic selective forces can drive the evolution of longevity regulation mechanisms within ecosystems, we carried out a three-step selection of long-lived yeast species by a lasting exposure to LCA under laboratory conditions.

**FIGURE 1:**
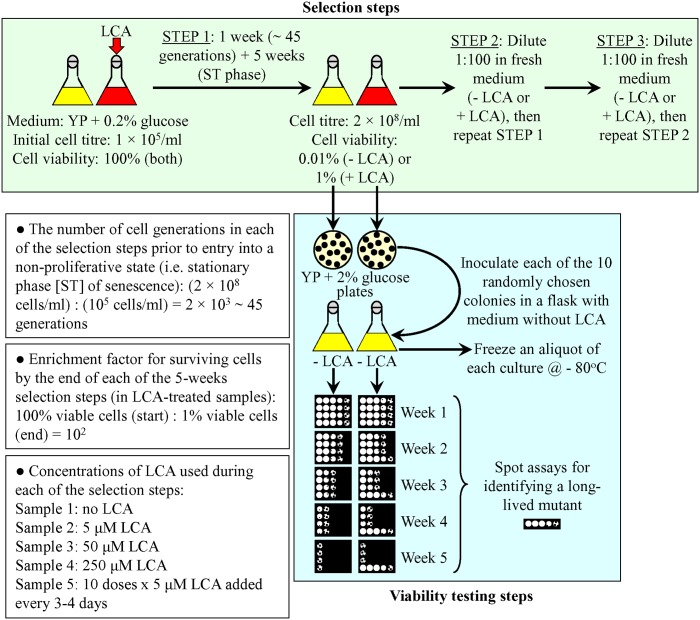
**A three-step process for selection of long-lived yeast species by a lasting exposure to LCA under laboratory conditions.** For each of the 5 weeks-long selection steps, the number of cell generations and enrichment factor for surviving cells were calculated as shown. The genomes of some of the cells surviving a selection step may contain mutations that extend yeast chronological lifespan (CLS). The genomes of other cells surviving a selection step may not undergo any changes affecting their CLS; the survival of these cells may have been caused only by the ability of LCA to extend yeast CLS. LCA concentrations used during each selection step are indicated. See text for details. LCA, lithocholic acid; ST, stationary growth phase; YP, medium containing 1% yeast extract and 2% peptone.

For the first step of such selection, yeast cells from an overnight culture were inoculated into a fresh, nutrient-rich YP medium (with or without LCA) containing 0.2% glucose to the initial cell titer of 1 × 10^5^ cells/ml (**Figure 1**). We chose 0.2% as the initial concentration of glucose in liquid medium for the LCA-driven selection of long-lived yeast species because the longevity-extending efficiency of LCA under caloric restriction (CR) on 0.2% glucose exceeds that under non-CR conditions on 2% glucose, a concentration typically used to culture yeast under laboratory conditions (Goldberg et al., 2009, 2010b; Arlia-Ciommo et al., 2014a). After 1 week of the incubation in this medium and following approximately 45 cell generations, yeast entered into a non-proliferative state of quiescence by reaching stationary (ST) growth phase at the cell titer of 2 × 10^8^ cells/ml (**Figure 1**). Following their entry into a non-proliferative state, yeast cells were cultured for additional 5 weeks. By the end of this cultivation, only ∼0.01% of cells in the medium without LCA remained viable. In the cell cultures that were supplemented with LCA, ∼1% of cells remained viable—due to the ability of LCA to increase the CLS of non-dividing (quiescent) yeast cells. Thus, the enrichment factor for cells surviving each selection step in LCA-treated samples was 10^2^ (**Figure 1**). According to our hypothesis of the hormetic selective forces driving the evolution of longevity regulation mechanisms, the genomes of some of the cells surviving a selection step may contain mutations that extend yeast CLS. Noteworthy, it is conceivable that the genomes of other cells surviving a selection step may not undergo any changes affecting their CLS; the survival of these cells may have been caused only by the ability of LCA to extend yeast CLS. The following five cultures were used to carry out the selection of long-lived yeast species: the first culture lacked LCA; the second culture contained 5 μM LCA added at the moment of cell inoculation; the third culture contained 50 μM LCA added at the moment of cell inoculation; the fourth culture contained 250 μM LCA added at the moment of cell inoculation; and the fifth culture was supplemented with 10 doses of 5 μM LCA each, by adding a 5-μM dose of LCA every 3 or 4 days (**Figure 1**). Of note, LCA exhibited the greatest longevity-extending effect in chronologically aging yeast if used at a final concentration of 50 μM (Goldberg et al., 2010b). At the end of the first selection step, aliquots of each of the five cultures were plated onto plates with solid YP medium containing 2% glucose. After 2 days, each of the 10 randomly chosen colonies was inoculated into a liquid medium without LCA to carry out a viability testing step (**Figure 1**). Every week, an aliquot of each culture was used for a spot assay of cell viability to identify long-lived mutants (if any) induced due to a lasting exposure of yeast to LCA during the selection step (**Figure 1**). If such mutants were detected, an aliquot of their culture in medium lacking LCA was frozen at -80°C.

To conduct the second selection step, an aliquot of the culture recovered at the end of the first step of selection was diluted 100-folds in a fresh YP medium containing 0.2% glucose, with or without LCA; if added to a culture for the second selection step, LCA was present at the same final concentration as that in the medium used for the first step of selection (**Figure 1**). From that point, the second selection step was carried out exactly as the first one and was followed by a spot-assay viability step described above (**Figure 1**). The second selection step was followed by the third selection step and then by a spot-assay viability step, both conducted as described previously (**Figure 1**).

**FIGURE 2:**
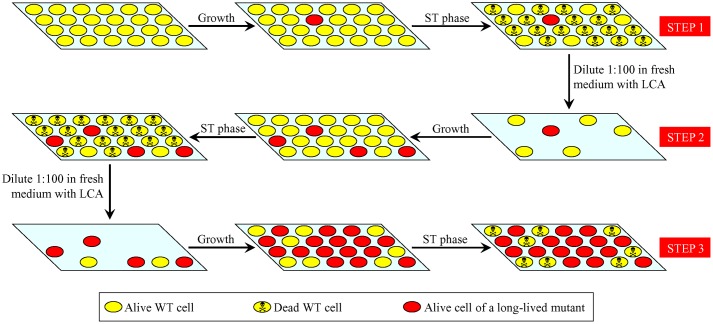
**Changes in the percentage of long-lived mutants within a population of yeast during three consecutive steps of the LCA-driven evolution of yeast species that live longer.** The percentage of such long-lived mutants in a population of yeast exposed to LCA is increased by the end of each selection step. Each next selection step yields the higher percentage of such mutants than the previous one. See text for details. LCA, lithocholic acid; ST, stationary growth phase; WT, wild-type.

The three-step selection of long-lived yeast species depicted in **Figure 1** was expected to increase the percentage of such mutant species within a population by the end of each selection step (**Figure 2**). Moreover, each next selection step was anticipated to yield the higher percentage of such mutants than the previous one (**Figure 2**; **Table 1**).

**Table 1:** The fraction of long-lived mutants in a population of yeast is expected to be increased by the end of each of the three consecutive steps during the LCA-driven selection of yeast species that live longer.

If the frequency of mutation is:	Enrichment factor for long-lived mutants	The fraction of long-lived mutants and their number in a population of yeast by the end of each of the three consecutive steps of LCA-driven experimental evolution	
		Fraction	Number
10^-8^ per generation	10^2^	Step 1: [10^-8^ × 45 generations (gen.)] × 10^2^ = 4.5 × 10^-5^	4–5 out of 100,000
		Step 2: [(4.5 × 10^-5^) + (10^-8^ × 45 gen.)] × 10^2^ ≈ 4.5 × 10^-3^	4–5 out of 1,000
		Step 3: [(4.5 × 10^-3^) + (10^-8^ × 45 gen.)] × 10^2^ ≈ 4.5 × 10^-1^	4–5 out of 10
10^-7^ per generation	10^2^	Step 1: (10^-7^ × 45 gen.) × 10^2^ = 4.5 × 10^-4^	4–5 out of 10,000
		Step 2: [(4.5 × 10^-4^) + (10^-7^ × 45 gen.)] × 10^2^ ≈ 4.5 × 10^-2^	4–5 out of 1,000
		Step 3: [(4.5 × 10^-2^) + (10^-7^ × 45 gen.)] × 10^2^ ≈ 4.5 × 10^0^	All
10^-6^ per generation	10^2^	Step 1: (10^-6^ × 45 gen.) × 10^2^ = 4.5 × 10^-3^	4–5 out of 1,000
		Step 2: [(4.5 × 10^-3^) + (10^-6^ × 45 gen.)] × 10^2^ ≈ 4.5 × 10^-1^	4–5 out of 10
		Step 3: [(4.5 × 10^-1^) + (10^-6^ × 45 gen.)] × 10^2^ ≈ 4.5 × 10^1^	All

*For each selection step, the number of cell generations and enrichment factor for long-lived mutants were calculated as shown in **Figure 1**.*

**FIGURE 3:**
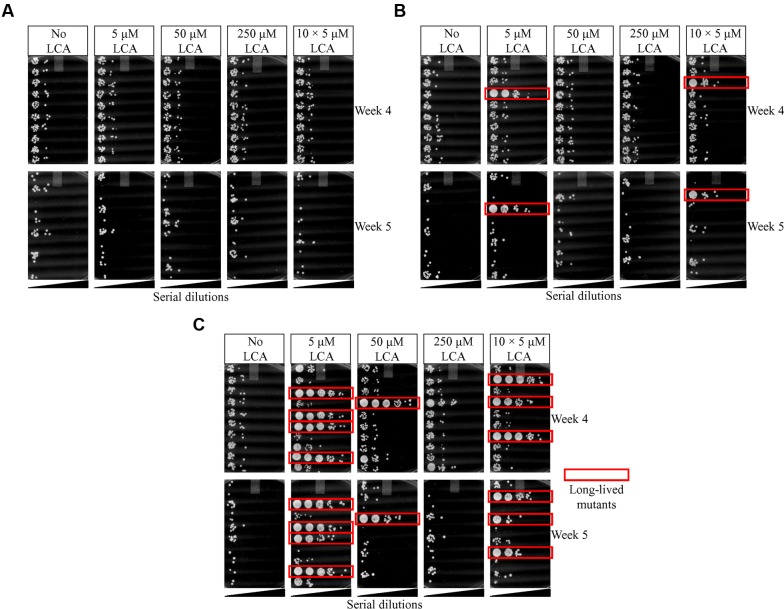
**Spot-assays of cell survival for each of the three consecutive steps of the LCA-driven selection of long-lived yeast species under laboratory conditions.** The selection steps were carried out as outlined in **Figures 1** and **2**. **(A)** No long-lived mutant yeast species were found at the end of the first selection step. **(B)** Four long-lived mutant yeast species were recovered at the end of the second selection step. **(C)** Sixteen long-lived mutant yeast species were recovered at the end of the third selection step.

By carrying out this three-step selection of long-lived yeast species under laboratory conditions, we found no such mutant species at the end of the first selection step, four long-lived mutants at the end of the second selection step, and 16 long-lived mutants at the end of the third selection step (**Figures 3A–C**, respectively). We therefore concluded that a long-term exposure of WT yeast to LCA during three consecutive selection steps yielded yeast species that live longer in the absence of LCA than their ancestor.

Our findings revealed the following order of different LCA concentrations ranked by the efficiency with which they cause the appearance of long-lived species (the frequencies of such appearances are shown in parentheses; they were calculated as detailed in **Table 1**): 5 μM LCA (≈4 × 10^-8^ per generation) > 10 doses × 5 μM LCA (≈3 × 10^-8^ per generation) > 50 μM LCA (≈1 × 10^-8^ per generation) > 250 μM LCA (no long-lived species found). Because the lowest used concentration of LCA resulted in the highest frequency of long-lived species appearance, it is unlikely that the longevity-extending mutations they carry are due to mutagenic action of LCA. Thus, these mutations are likely to arise spontaneously in yeast populations subjected to a lasting exposure to LCA.

### Three Selected Long-Lived Yeast Species Can Maintain Their Greatly Extended Lifespans Following Numerous Passages in Medium without LCA

**FIGURE 4:**
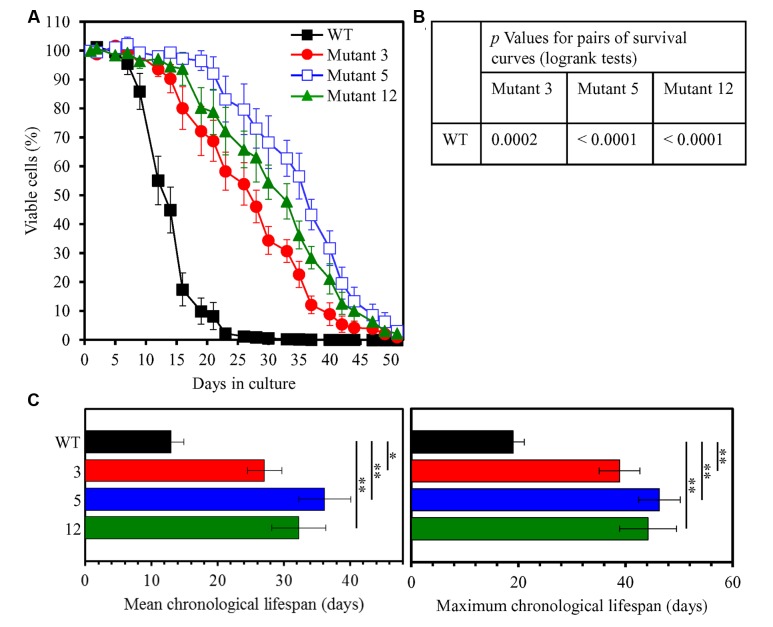
**The selected long-lived yeast species 3, 5, and 12 maintain their ability to live much longer than WT following five successive passages in medium lacking LCA.** Long-lived mutants 3, 5, and 12 underwent five consecutive passages in liquid medium without LCA. Each of them was then inoculated into liquid YP medium lacking LCA and initially containing 0.2% glucose. **(A)** Survival curves of chronologically aging WT and long-lived mutant strains cultured in this medium are shown. Data are presented as means ± SEM (*n* = 3). **(B)** *p* Values for pairs of survival curves of the WT and mutant strains. Survival curves shown in **(A)** were compared. The survival curve for the WT strain was considered statistically different from the survival curve for the mutant strain if the *p* value was less than 0.05. The *p* values for comparing pairs of survival curves using the log-rank test were calculated as described in Section “Materials and Methods.” **(C)** Survival curves shown in **(A)** were used to calculate the mean and maximum chronological lifespans for WT and mutant strains. Data are presented as means ± SEM (*n* = 3; ^∗^*p* < 0.05; ^∗∗^*p* < 0.01).

The three-step experimental evolution of long-lived yeast species by a prolonged exposure of WT yeast to LCA resulted in selection of 20 species that in a spot-assay lived longer than their ancestor in medium lacking LCA (**Figure 3**). An aliquot of the culture of each of these long-lived yeast species was frozen at -80°C immediately after being recovered during the second or third selection step. To test the abilities of these species to maintain their considerably increased lifespans during the first passage in medium without LCA, each aliquot was thawed and then inoculated into liquid YP medium lacking this bile acid and initially containing 0.2% glucose. Our comparative analysis of the CLS of WT strain and all of these selected long-lived yeast species revealed that each of the 20 species maintains its ability to live longer than WT during the first passage in liquid medium (Supplementary Figure S1). Yeast species 3, 5, and 12 exhibited the highest extent of longevity extension during the first passage in medium without LCA (Supplementary Figure S1). These three yeast species underwent four more passages (each carried out as described for the first passage) in liquid medium without LCA; they then were tested again for their abilities to maintain greatly extended lifespans, now following five successive passages in medium without LCA. As we found, each of the three extremely long-lived yeast species evolved under laboratory conditions sustains its significantly prolonged CLS after five passages in medium without LCA (**Figure 4**).

### The Extended Longevity of Each of the Three Selected Long-Lived Yeast Mutants Is a Dominant Polygenic Trait

**FIGURE 5:**
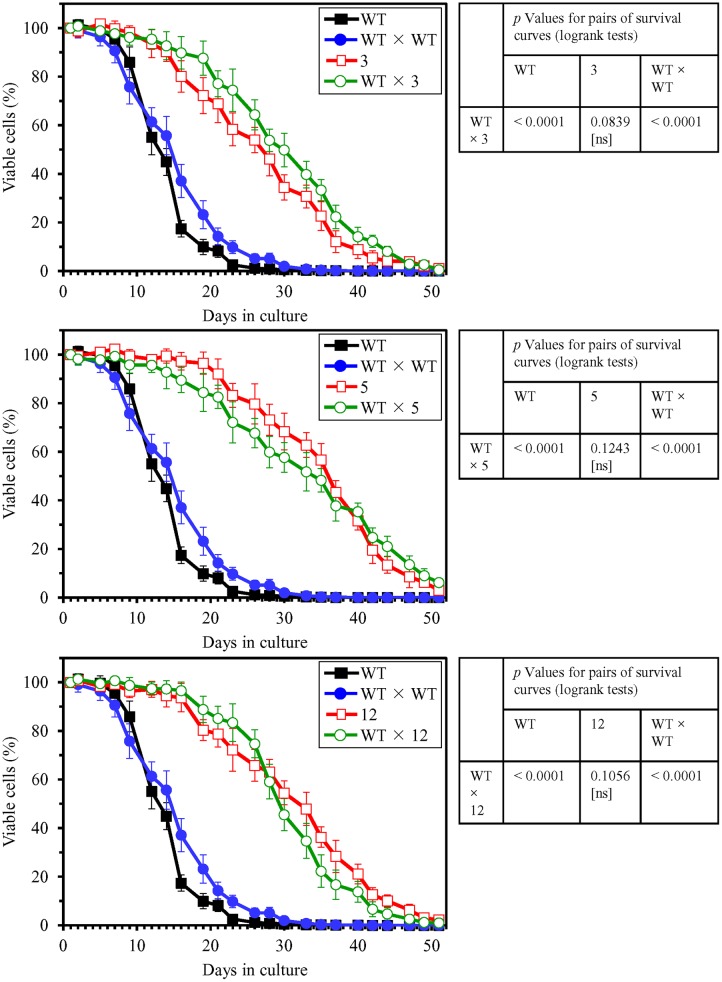
**The extended longevity of each of the three long-lived yeast mutants evolved under laboratory conditions is a dominant genetic trait.** The parental haploid WT strain BY4742, the WT × WT diploid strain formed by mating between the haploid WT strains BY4741 and BY4742, the long-lived mutant strains 3, 5, and 12 (each in the BY4742 genetic background), and the WT × 3, WT × 5, and WT × 12 diploid strains were cultured in YP medium without LCA initially containing 0.2% glucose. Survival curves of chronologically aging cells are shown. Data are presented as means ± SEM (*n* = 4). The *p* values for comparing pairs of survival curves using the log-rank test were calculated as described in Section “Materials and Methods.” The survival curve for the WT × 3, WT × 5, or WT × 12 diploid strain was considered statistically different from the survival curve for the parental haploid mutant strain, parental haploid WT strain, or WT × WT diploid strain if the *p* value was less than 0.05. *ns*, not significant.

**FIGURE 6:**
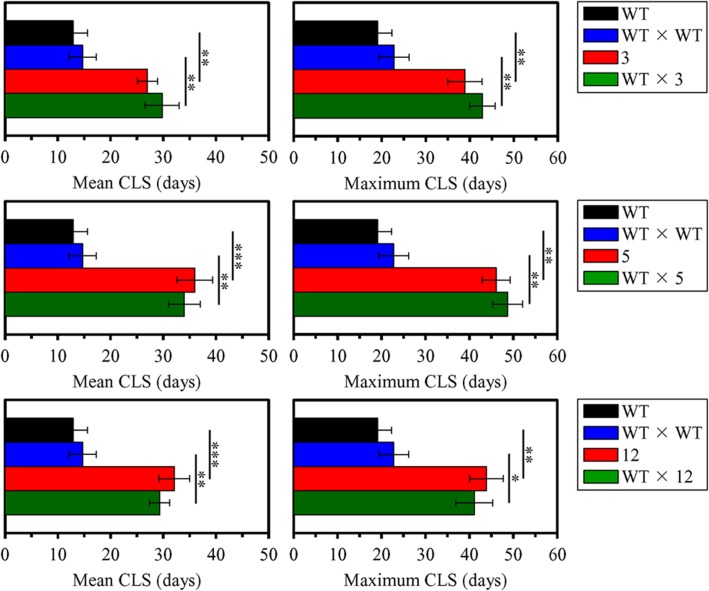
**The extended longevity of each of the three long-lived yeast mutants evolved under laboratory conditions is a dominant genetic trait.** The parental haploid WT strain BY4742, the WT × WT diploid strain formed by mating between the haploid WT strains BY4741 and BY4742, the long-lived mutant strains 3, 5, and 12 (each in the BY4742 genetic background), and the WT × 3, WT × 5, and WT × 12 diploid strains were cultured in YP medium without LCA initially containing 0.2% glucose. Survival curves shown in **Figure 7** were used to calculate the mean and maximum chronological lifespans for WT and mutant strains. Data are presented as means ± SEM (*n* = 4; ^∗^*p* < 0.05; ^∗∗^*p* < 0.01; ^∗∗∗^*p* < 0.001).

Each of the three selected long-lived yeast mutants (each of mating type α and in the BY4742 genetic background, i.e., *MATα his3Δ1 leu2Δ0 lys2Δ0 ura3Δ0*) was subjected to the backcross mating with the WT strain BY4741 (*MATa his3Δ1 leu2Δ0 met15Δ0 ura3Δ0*) of opposite mating type. A WT × WT diploid strain was created by mating between the haploid WT strains BY4741 (*MATa his3Δ1 leu2Δ0 met15Δ0 ura3Δ0*) and BY4742 (*MATα his3Δ1 leu2Δ0 lys2Δ0 ura3Δ0*). We compared the CLS of each, the WT × 3, WT × 5, and WT × 12 diploid strain, to those of the parental haploid mutant strain, parental haploid WT strain and WT × WT diploid strain; yeast cells in these experiments were cultured in YP medium lacking LCA and initially containing 0.2% glucose. We found that chronologically aging cells of the WT × 3, WT × 5, and WT × 12 diploid strains live (1) almost as long as chronologically aging cells of the parental haploid mutant strain; and (2) significantly longer than chronologically aging cells of the WT × WT diploid strain and the parental haploid WT strain (**Figure 5**). Both the mean and maximum CLS of each, the WT × 3, WT × 5, and WT × 12 diploid strain, exceeded those of the WT × WT diploid strain to a similar degree as the mean and maximum CLS of the parental haploid mutant strains exceeded those of the parental haploid WT strain (**Figure 6**). Based on these observations, we concluded that the extended longevity of each of the three long-lived yeast mutants is a dominant genetic trait.

To elucidate if the extended longevity of each of the three long-lived yeast mutants is a monogenic or polygenic genetic trait, we first subjected the WT × 3, WT × 5, and WT × 12 diploid strains to sporulation. We then removed the outer cell wall surrounding the four ascospores within individual asci by treatment with Zymolyase, and used micromanipulation to separate these ascospores. For each of the three sporulated diploids, we conducted tetrad analysis of ascospores from six randomly chosen asci (i.e., the four ascospores recovered within each ascus). We compared the CLS of individual meiotic segregants originated from the WT × 3, WT × 5, and WT × 12 diploid strains to the CLS of the parental mutant and WT strains. This comparative analysis revealed the following: (1) chronologically aging cells of all four ascospores within each of the six tested tetrads exhibit an extended CLS characteristic of the parental mutant strain and live significantly longer than cells of the parental WT strain; and (2) this relationship between the CLS of individual meiotic segregants, the parental mutant strain and the parental WT strain is observed for each of the three diploid strains, namely WT × 3, WT × 5, and WT × 12 (Supplementary Figures S2–S10). The observed relationship between the CLS of individual meiotic segregants, the parental mutant strain and the parental WT strain differed from a relationship that was expected if the extended longevity of a long-lived yeast mutant evolved under laboratory conditions was due to a single-gene mutation or mutations in two nuclear genes. Indeed, if the trait of extended longevity was monogenic, it was anticipated that two of the four ascospores within each of the six randomly chosen tetrads do not exhibit an extended CLS characteristic of the parental mutant strain and live almost as short life as cells of the parental WT strain (Sherman, 2002; Amberg et al., 2005; Boone et al., 2016). Furthermore, if the trait of extended longevity was caused by dominant mutations in two nuclear genes, it was projected that one or two of the four ascospores within three, four, or five out of the six randomly chosen tetrads do not exhibit an extended CLS characteristic of the parental mutant strain and live almost as short life as cells of the parental WT strain (Sherman, 2002; Amberg et al., 2005; Boone et al., 2016). Based on these observations, we concluded that the extended longevity of each of the three selected long-lived yeast mutants is a polygenic genetic trait caused by mutations in more than two nuclear genes.

### Each of the Three Selected Long-Lived Yeast Mutants Exhibits Altered Age-Related Chronology of Mitochondrial Respiration and Enhanced Resistance to Chronic Stresses

**FIGURE 7:**
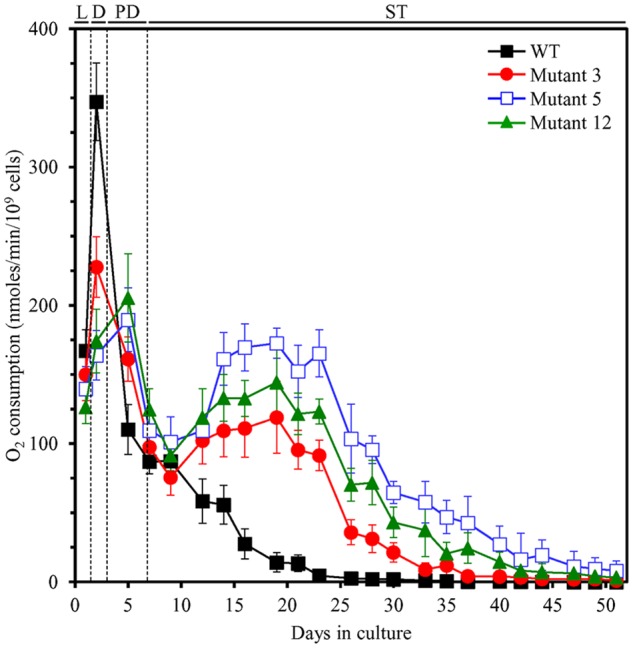
**Each of the three selected long-lived yeast mutants exhibits altered age-related chronology of mitochondrial respiration.** The parental haploid WT strain and the long-lived mutant strains 3, 5, and 12 were cultured in YP medium without LCA initially containing 0.2% glucose. The dynamics of age-dependent changes in the rate of oxygen consumption by chronologically aging yeast is shown. Data are presented as means ± SEM (*n* = 3). D, diauxic growth phase; L, logarithmic growth phase; PD, post-diauxic growth phase; ST, stationary growth phase.

**FIGURE 8:**
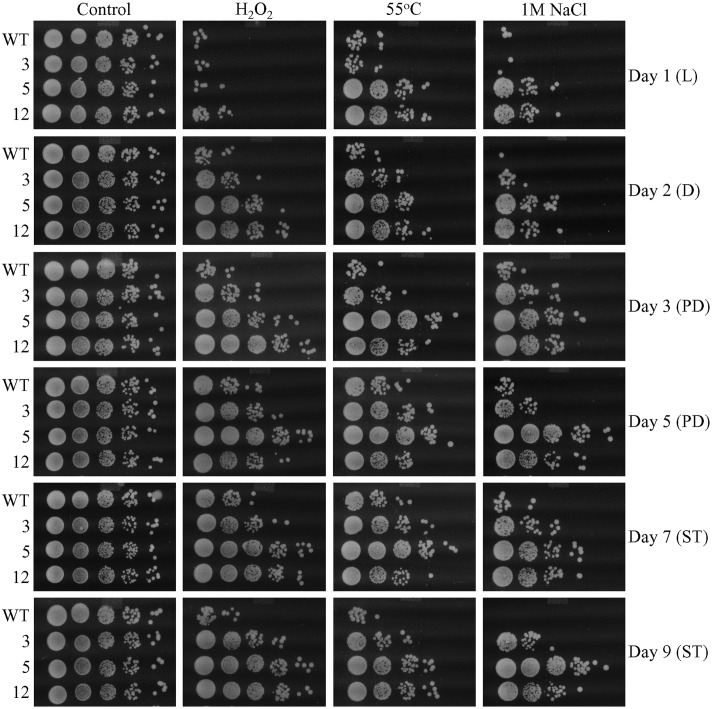
**Each of the three selected long-lived yeast mutants displays enhanced resistance to chronic oxidative, thermal, and osmotic stresses.** The parental haploid WT strain and the long-lived mutant strains 3, 5, and 12 were cultured in YP medium without LCA initially containing 0.2% glucose. Cell aliquots were recovered from various growth phases. The resistance of yeast to chronic oxidative, thermal, and osmotic stresses was monitored as described in Section “Materials and Methods.” D, diauxic growth phase; L, logarithmic growth phase; PD, post-diauxic growth phase; ST, stationary growth phase.

One of the key aspects of our hypothesis of the hormetic selective forces driving the evolution of longevity regulation mechanisms is an assumption that all yeast species within an ecosystem can respond to LCA and other bile acids released into the ecosystem by animals (Goldberg et al., 2010a,b; Burstein et al., 2012a). This response consists in the ability of yeast to develop mechanisms of protection against cellular damage caused by the mildly toxic molecules of bile acids (Goldberg et al., 2010a,b; Burstein et al., 2012a). Of note, our analysis of how different concentrations of LCA impact yeast longevity has revealed that this bile acid delays yeast chronological aging by eliciting a hormetic stress response (Goldberg et al., 2010b; Burstein et al., 2012a), which is characterized by a non-linear and biphasic dose–response curve (Goldberg et al., 2010a; Calabrese and Mattson, 2011; Burstein et al., 2012a; Calabrese et al., 2012; Leonov et al., 2015; Lutchman et al., 2016). Our recent studies also demonstrated that the ability of LCA to elicit such longevity-extending stress response is due in part to alterations in mitochondrial functionality caused by LCA, including changes in the age-related chronology of mitochondrial respiration (Goldberg et al., 2010b; Burstein et al., 2012b; Beach et al., 2013, 2015a,b; Arlia-Ciommo et al., 2014a,b; Burstein and Titorenko, 2014; Medkour and Titorenko, 2016). These LCA-driven changes in mitochondrial respiration allow mitochondria to establish and sustain an aging-delaying pattern of the entire cell (Goldberg et al., 2010b; Burstein et al., 2012b; Beach et al., 2013, 2015a,b; Arlia-Ciommo et al., 2014a,b; Burstein and Titorenko, 2014; Medkour and Titorenko, 2016). Enhanced resistance of chronologically aging yeast to chronic oxidative, thermal, and osmotic stresses is part of such LCA-driven cellular pattern (Goldberg et al., 2010b; Burstein et al., 2012b; Beach et al., 2015b; Medkour and Titorenko, 2016); this kind of mitochondria-dependent hormetic stress response is called “mitohormesis” (Tapia, 2006; Schulz et al., 2007; Ristow and Zarse, 2010; Ristow, 2014; Yun and Finkel, 2014). We thought, therefore, that some of the three long-lived yeast mutants selected under laboratory conditions may have evolved an altered pattern of mitochondrial respiration and/or stress susceptibility. We found that in WT yeast grown in YP medium with 0.2% glucose in the absence of LCA, the rate of oxygen consumption by mitochondria was (1) greatly amplified when yeast entered diauxic (D) growth phase that begins on day 2 of culturing; (2) sharply declined during the subsequent post-diauxic (PD; occurs between days 3 and 7 of culturing) phase of growth; and (3) gradually reduced during ST (begins after day 7 of culturing) growth phase that follows PD phase (**Figure 7**). In contrast, in all three selected long-lived yeast mutants grown in this medium, the rate of oxygen consumption by mitochondria was (1) amplified to a significantly lesser extent during D phase than it was in WT yeast; (2) not declined as sharply during the subsequent PD phase of growth as it was in WT yeast; and (3) elevated again for a long period of time during ST phase (to the level similar to that seen in these yeast species during PD phase) and began to decline slowly only deep into ST phase—unlike the situation seen in WT yeast (**Figure 7**). Moreover, we found that each of the three selected long-lived yeast mutants grown in this medium displays enhanced cell resistance to chronic oxidative, thermal, and osmotic stresses, especially during D, PD, and ST growth phases (**Figure 8**).

## Discussion

Our previously proposed hypothesis posits that LCA and other bile acids released into the environment by animals may represent interspecies chemical signals capable of generating selective pressure for the evolution of longevity regulation mechanisms in yeast (Goldberg et al., 2010a; Burstein et al., 2012a). In this study, we empirically validated our hypothesis by being able to model the LCA-driven evolution of long-lived yeast species under laboratory conditions. To carry out such experimental evolution, cells of WT strain were subjected to a three-step process of a lasting exposure to exogenously added LCA. Three out of 20 long-lived mutants selected during this evolution process were able to maintain the trait of substantially extended CLS after numerous passages in medium without LCA or other bile acid. Our findings imply that the prolonged longevity of each of the three long-lived yeast species is a dominant polygenic trait caused by mutations in more than two nuclear genes.

Noteworthy, we cannot rule out the possibility that the longevity extension of one or more of these long-lived yeast species is caused by some transgenerationally inherited epigenetic changes, rather than by DNA sequence mutations. Indeed, recent evidence indicates that various environmental, dietary, genetic, and pharmacological interventions elicit certain epigenetic changes that can influence the aging process and define longevity in evolutionarily distant eukaryotic organisms (Dang et al., 2009; Greer et al., 2010; Li et al., 2011; Moskalev et al., 2014; Benayoun et al., 2015; Li and Casanueva, 2016; Pal and Tyler, 2016; Sen et al., 2016). Some of these epigenetic changes can be epigenetically inherited for several generations, thus they can act in a transgenerational manner to affect the rate of aging and define the lifespan of the offspring (Greer et al., 2011; Benayoun et al., 2015; Li and Casanueva, 2016; Pal and Tyler, 2016).

It seems implausible that the longevity extension of one or more of the three long-lived yeast species is caused by dominant mitochondrially inherited mutations. Indeed, unlike higher eukaryotes that inherit mitochondrial DNA (mtDNA) uniparentally, mtDNA of the yeast *S. cerevisiae* is known to be inherited in a biparental manner which is independent of mating type (Griffiths et al., 2000; Solieri, 2010). Thus, crosses of WT yeast cells with yeast strains carrying various so-called *mit* mutations in mtDNA yield heteroplasmic zygotes displaying WT phenotype (Griffiths et al., 2000; Solieri, 2010). Furthermore, a mitotic division of these heteroplasmic zygotes produces only diploid buds having WT phenotype (Griffiths et al., 2000). Moreover, all four meiotic ascospores within individual tetrads recovered after sporulation of such diploid buds also exhibits only WT phenotype (Griffiths et al., 2000; Solieri, 2010). In case of mutations in yeast mtDNA which lead to erythromycin resistance (*ery^R^*), a mitotic division of the original zygotic heteroplasmon results in the formation of diploid buds some of which are *ery^R^*, whereas others are sensitive to erythromycin (*ery^S^*) (Griffiths et al., 2000). Sporulation of *ery^R^* diploid buds yields only *ery^R^* meiotic ascospores, whereas sporulation of diploid buds that are *ery^S^* produces only *ery^S^* meiotic ascospores (Griffiths et al., 2000). Therefore, the inheritance patterns for each of the three long-lived yeast mutants selected in this study differ from the inheritance patterns of the *mit* and *ery^R^* groups of mutations in mtDNA; mutations that belong to these two groups are known to be the most common mutational lesions in yeast mtDNA (Griffiths et al., 2000). It needs to be mentioned that one inheritance pattern for each of the three long-lived yeast mutants selected in this study is similar to that for yeast mutants carrying suppressive or hyper-suppressive petite mutations in mtDNA. Specifically, crosses of WT yeast cells with yeast strains carrying these mtDNA mutations yield heteroplasmic zygotes that are mainly or almost exclusively exhibit the petite phenotype of deficiency in mitochondrial respiration (MacAlpine et al., 2001; Solieri, 2010). However, unlike diploids formed by mating between the WT strain and any of the three selected long-lived yeast mutants, these respiration-deficient petite zygotes cannot sporulate (Horecker and Stadtman, 2016).

Our hypothesis further predicted that, if a long-term exposure to exogenous LCA will yield long-lived yeast species, these mutant species may evolve an altered pattern of mitochondrial respiration and/or stress susceptibility. In confirmation of this prediction, we found that each of the three selected long-lived mutants of yeast exhibits altered age-related chronology of mitochondrial respiration and enhanced resistance to chronic oxidative, thermal, and osmotic stresses. In sum, these findings provide evidence in support of our hypothesis of the hormetic selective forces that drive the evolution of longevity regulation mechanisms within ecosystems.

In the future, it would be interesting to use the long-lived mutant strains selected in this study for empirical verification of two groups of evolutionary theories of aging. One group of these theories is based on the concept of programmed aging; these theories are trying to explain how the evolutionary force actively limits organismal lifespan at an age unique to each species (Skulachev, 1999a,b, 2001, 2002a,b; Longo et al., 2005; Skulachev and Longo, 2005; Severin et al., 2008; Goldsmith, 2011, 2012, 2014; Mitteldorf, 2012; Trindade et al., 2013). Another group of evolutionary aging theories is based on the notion of non-programmed aging; these theories are attempting to rationalize how lack of the evolutionary force passively limits organismal lifespan at an age characteristic of each species (Ljubuncic and Reznick, 2009; Goldsmith, 2011, 2012, 2014; Mitteldorf, 2012; Trindade et al., 2013). To perform the critical test for empirical verification of evolutionary theories of programmed or non-programmed aging, it would be necessary to investigate if the dominant polygenic trait extending longevity of each selected long-lived yeast mutant influences early-life fitness when each mutant grows and ages alone—i.e., in the absence of a parental WT strain. All evolutionary theories of non-programmed aging posit that under such conditions any longevity-extending genetic trait must reduce early-life fitness of an organism (Medawar, 1952; Williams, 1957; Kirkwood, 1977; Kirkwood and Holliday, 1979; Charlesworth, 2000, 2001; Kirkwood and Austad, 2000; Ljubuncic and Reznick, 2009; Goldsmith, 2011, 2012, 2014; Mitteldorf, 2012; Trindade et al., 2013). In contrast, all contemporary evolutionary theories of programmed aging assume that only those longevity-extending genetic traits that affect critical for early-life fitness modules of the pro-aging signaling network could reduce such fitness, whereas the longevity-extending genetic traits that affect other modules of such signaling network are unlikely to have an effect on organismal early-life fitness (Kenyon et al., 1993; Parsons, 1995; Zwaan et al., 1995; Brown-Borg et al., 1996; Partridge et al., 1999; Buck et al., 2000; Stearns et al., 2000; Clancy et al., 2001; Tatar et al., 2001; Marden et al., 2003; Liu et al., 2005; Longo et al., 2005; Partridge and Gems, 2006; Giannakou et al., 2007; Delaney et al., 2011). The following key traits of early-life fitness need to be assessed to conduct such test for empirical validation of two groups of evolutionary theories of aging: (1) the exponential growth rates of yeast cells in media containing a fermentable or non-fermentable carbon source; (2) the efficacy of post-exponential growth of yeast cells in media containing a fermentable or non-fermentable carbon source; and (3) fecundity of yeast cells, which can be quantitatively assessed by monitoring the efficacies of their sexual reproduction by mating and sporulation.

Moreover, all evolutionary theories of programmed aging posit that the evolutionary force actively limits organismal lifespan at an age unique to each species (Skulachev, 1999a,b, 2001, 2002a,b; Longo et al., 2005; Skulachev and Longo, 2005; Severin et al., 2008; Ljubuncic and Reznick, 2009; Mitteldorf, 2010, 2012; Goldsmith, 2011, 2012, 2014; Trindade et al., 2013). All these theories are based on the premise that natural selection resulted in preferential reproduction of those members of various species that have evolved certain mechanisms for limiting their lifespans in a species-specific fashion and upon reaching a species-specific age (Libertini, 1988; Skulachev, 1997, 1999a,b, 2001; Longo et al., 2005; Goldsmith, 2008, 2011, 2012, 2014). This is because natural selection limits organismal lifespan at an age unique to each species by actively retaining only those alleles of pleiotropic genes that increase early-life fitness and thus reduce fitness at old age (Williams, 1957; Kirkwood, 1977; Kirkwood and Holliday, 1979; Kirkwood and Austad, 2000; Goldsmith, 2011, 2012, 2014). To perform the critical test for empirical validation of these evolutionary theories of programmed aging, it would be necessary to assess if the dominant polygenic trait extending longevity of each of the three selected yeast mutants influences the relative fitness of each mutant in a direct competition assay with the parental WT strain. Such direct competition assay mimics the process of natural selection within a mixed population of individuals that belong to the same species but exhibit different longevity-defining genetic backgrounds, have lifespans at a species-specific age and beyond it, and compete for nutrients and other environmental resources. If the evolutionary theories of programmed aging are valid, each of the three long-lived mutants is expected to be forced out of the laboratory ecosystem by the parental WT strain exhibiting shorter lifespan. If each long-lived mutant will exhibit reduced relative fitness in the direct competition assay with WT cells, the following conclusions can be made: (1) yeast cells may have evolved some mechanisms for limiting their lifespan upon reaching a certain chronological age; and (2) these mechanisms may drive the evolution of yeast longevity toward maintaining a finite yeast lifespan within ecosystems.

## Author Contributions

VT designed and supervised the study. AG-P, PK, MB, NA, FN, and TI performed experiments and analyzed the data. AG-P, PK, and VT wrote the manuscript. All authors read the final version of the manuscript.

## Conflict of Interest Statement

The authors declare that the research was conducted in the absence of any commercial or financial relationships that could be construed as a potential conflict of interest.
