# Supplementary material for: Empirical Validation of a Hypothesis of the Hormetic Selective Forces Driving the Evolution of Longevity Regulation Mechanisms
Source: Front Genet. 2016 Dec 6;7:216. doi: 10.3389/fgene.2016.00216 (PMC5138192; doi:10.3389/fgene.2016.00216)
Supplement: Supplementary file 1 [file Data_Sheet_1.DOCX]

Supplementary Material

Empirical Validation of a Hypothesis of the Hormetic Selective Forces Driving the Evolution of Longevity Regulation Mechanisms

**Alejandra Gomez-Perez, Pavlo Kyryakov, Michelle T. Burstein, Nimara Asbah, Forough Noohi, Tatiana Iouk, Vladimir I. Titorenko***

*** Correspondence:** Vladimir I. Titorenko: [vladimir.titorenko@concordia.ca](mailto:vladimir.titorenko@concordia.ca)

**Supplementary Figures**


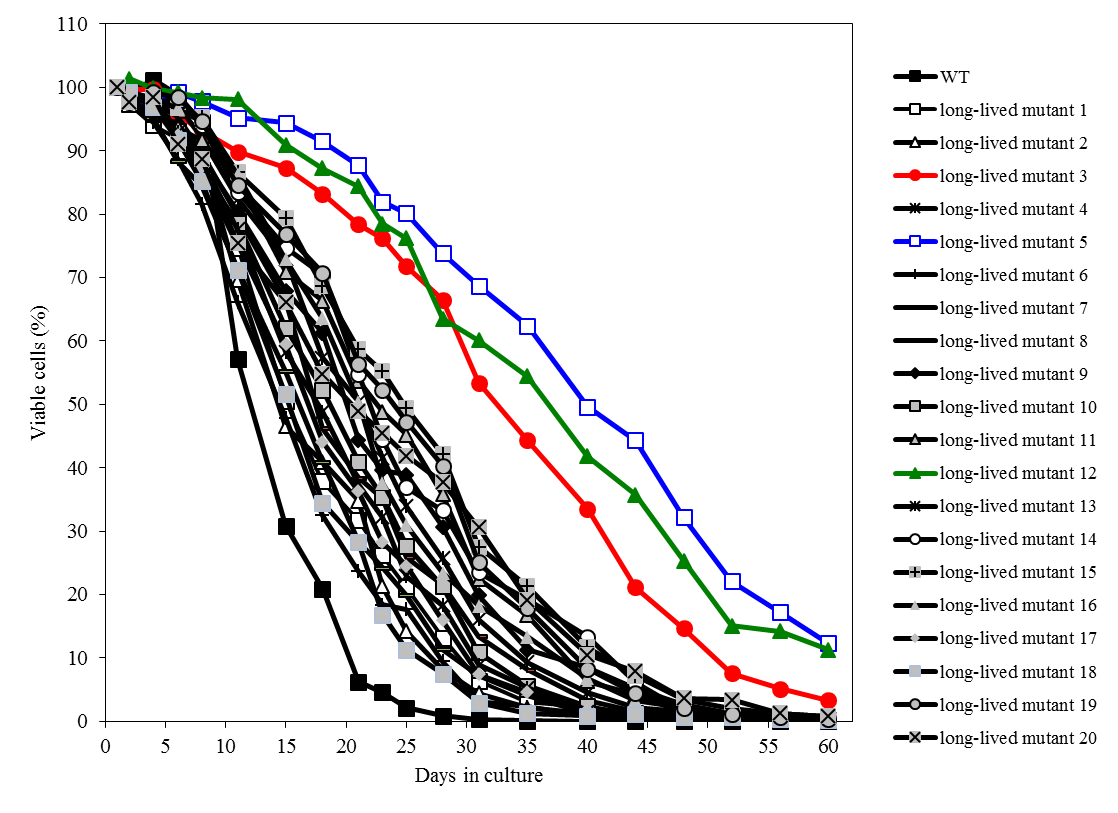


**Supplementary Figure 1.** **Each of the 20 selected long-lived yeast species maintains its ability to live longer than wild-type (WT) strain during their first passage in medium lacking LCA.** Yeast species 3, 5 and 12 exhibit the highest extent of longevity extension during such passage. An aliquot of the culture of each of the 20 selected long-lived yeast species (that were frozen at -80^o^C immediately after being recovered during the second or third selection step) was thawed and then inoculated into liquid YP medium lacking LCA and initially containing 0.2% glucose. Survival curves of chronologically aging WT and long-lived mutant strains cultured in this medium are shown. Dara are presented as means (n = 2).

**
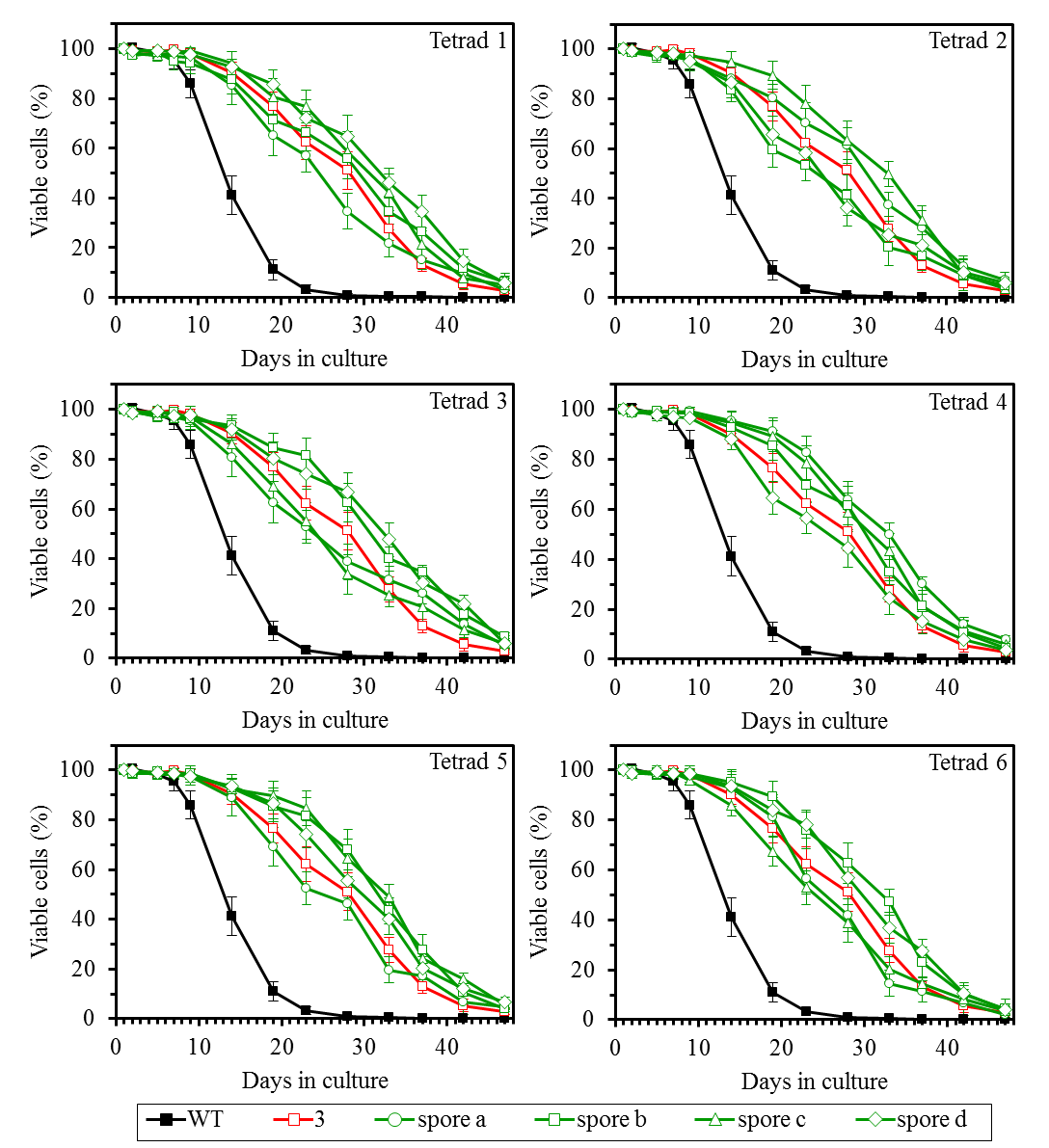
**

**Supplementary Figure 2.** **Chronologically aging cells of all four ascospores within each of the six randomly chosen tetrads that originated from the WT 🞨 3 diploid exhibit an extended CLS characteristic of the parental mutant strain 3 and live significantly longer than cells of the parental WT strain.** The parental WT strain BY4742, the long-lived parental mutant strain 3 (in the BY4742 genetic background) and the four ascospores recovered from each of the six randomly chosen tetrads were cultured in YP medium without LCA initially containing 0.2% glucose. Survival curves of chronologically aging cells are shown. Data are presented as means ± SEM (n = 6 for the parental WT strain; n = 2 for the parental mutant strain and for ascospores a, b, c and d).

**
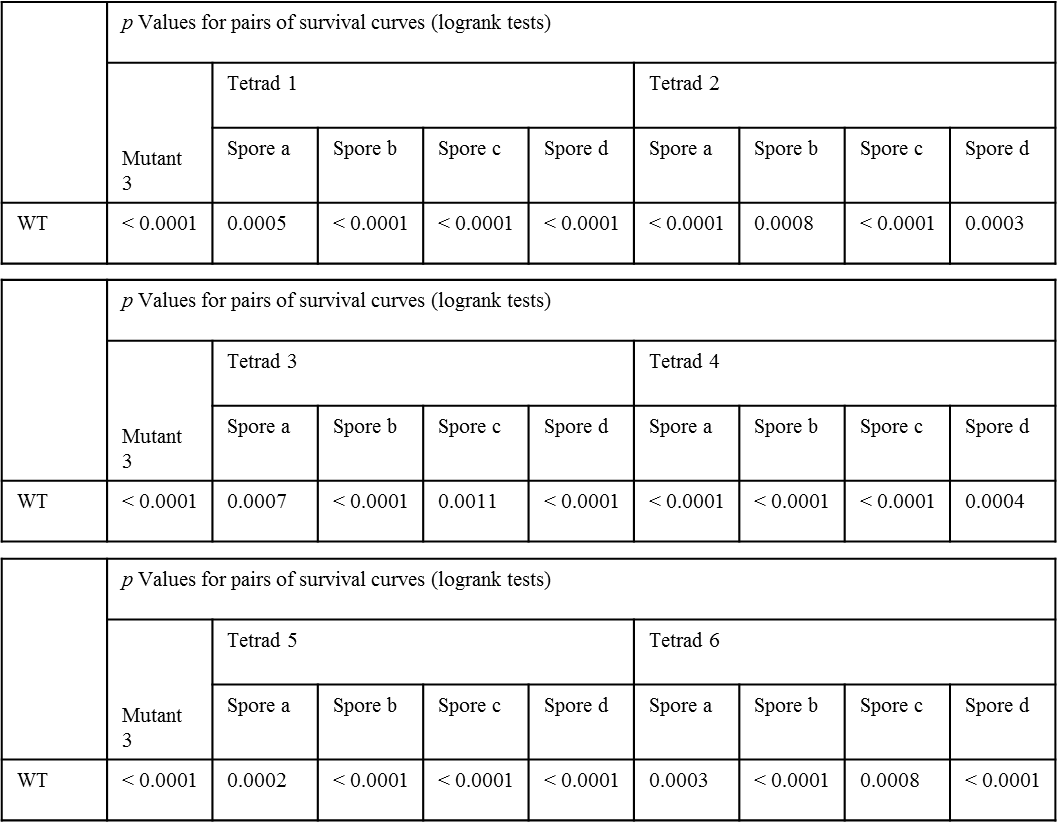
**

**Supplementary Figure 3.** **Chronologically aging cells of all four ascospores within each of the six randomly chosen tetrads that originated from the WT 🞨 3 diploid exhibit an extended CLS characteristic of the parental mutant strain 3 and live significantly longer than cells of the parental WT strain.** The parental WT strain BY4742, the long-lived parental mutant strain 3 (in the BY4742 genetic background) and the four ascospores recovered from each of the six randomly chosen tetrads were cultured in YP medium without LCA initially containing 0.2% glucose. Survival curves shown in Supplemental Figure 2 were used to calculate *p* values for various pairs of survival curves of different yeast strains. The survival curve for the WT strain was considered statistically different from the survival curve for the mutant strain 3 or from the survival curve for the ascospore if the *p* value was less than 0.05. The *p* values for comparing pairs of survival curves using the logrank test were calculated as described in Materials and Methods.


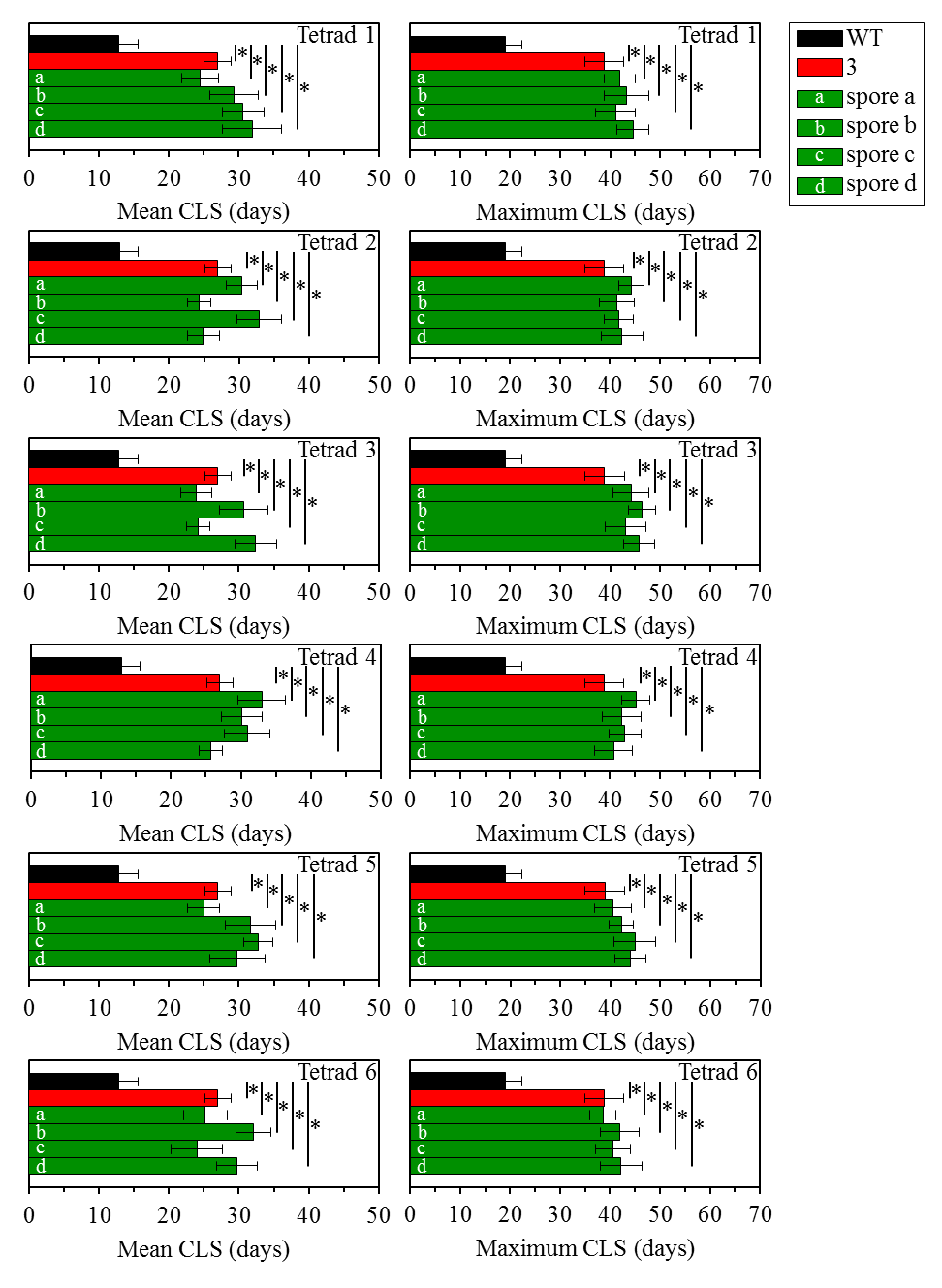

**Supplementary Figure 4.** **Chronologically aging cells of all four ascospores within each of the six randomly chosen tetrads that originated from the WT 🞨 3 diploid exhibit an extended CLS characteristic of the parental mutant strain 3 and live significantly longer than cells of the parental WT strain.** The parental WT strain BY4742, the long-lived parental mutant strain 3 (in the BY4742 genetic background) and the four ascospores recovered from each of the six randomly chosen tetrads were cultured in YP medium without LCA initially containing 0.2% glucose. Survival curves shown in Supplemental Figure 2 were used to calculate the mean and maximum CLS for different yeast strains. Data are presented as means ± SEM (n = 6 for the parental WT strain; n = 2 for the parental mutant strain and for ascospores a, b, c and d; *p < 0.05).


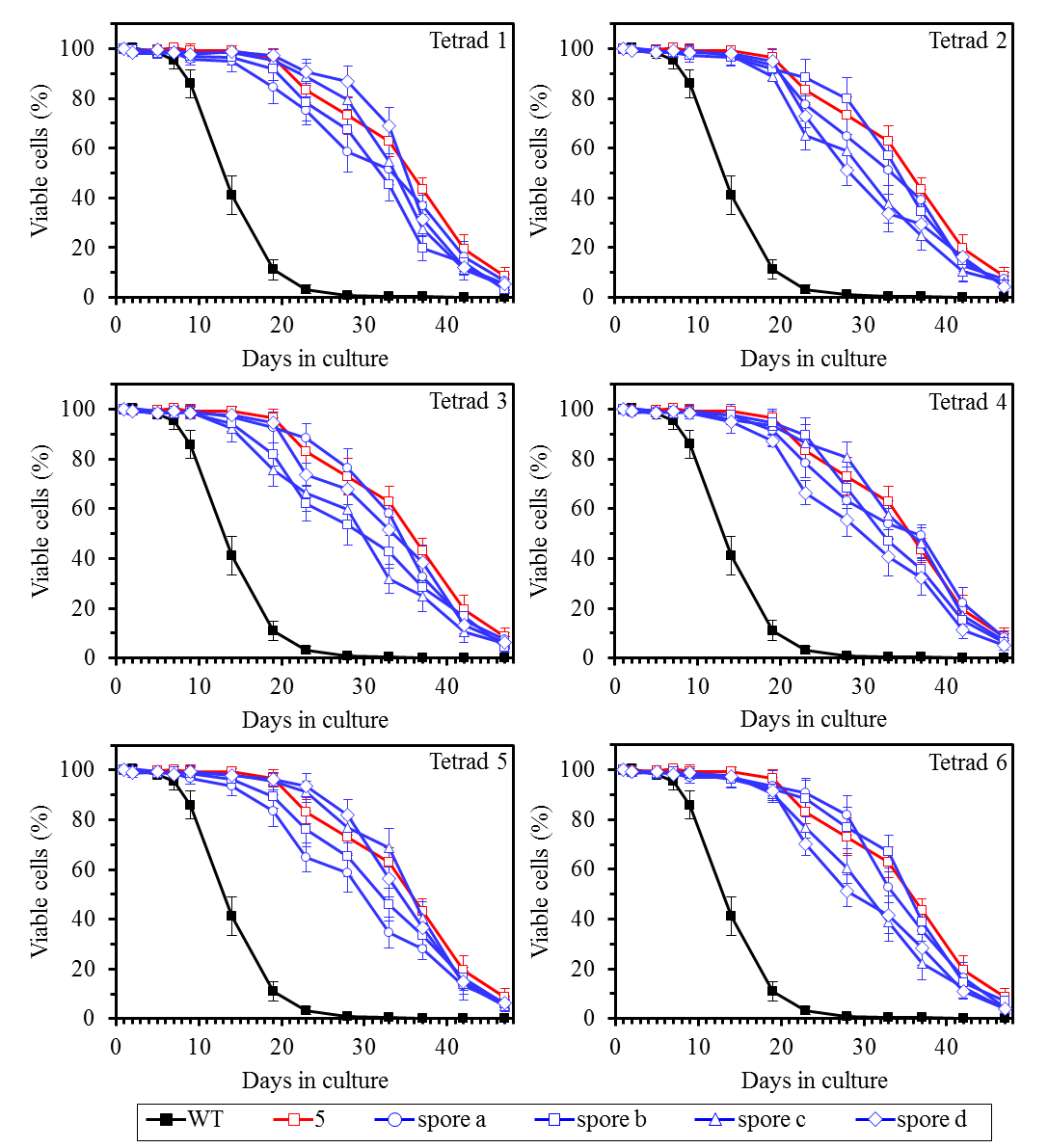


**Supplementary Figure 5.** **Chronologically aging cells of all four ascospores within each of the six randomly chosen tetrads that originated from the WT 🞨 5 diploid exhibit an extended CLS characteristic of the parental mutant strain 5 and live significantly longer than cells of the parental WT strain.** The parental WT strain BY4742, the long-lived parental mutant strain 5 (in the BY4742 genetic background) and the four ascospores recovered from each of the six randomly chosen tetrads were cultured in YP medium without LCA initially containing 0.2% glucose. Survival curves of chronologically aging cells are shown. Data are presented as means ± SEM (n = 6 for the parental WT strain; n = 2 for the parental mutant strain and for ascospores a, b, c and d).


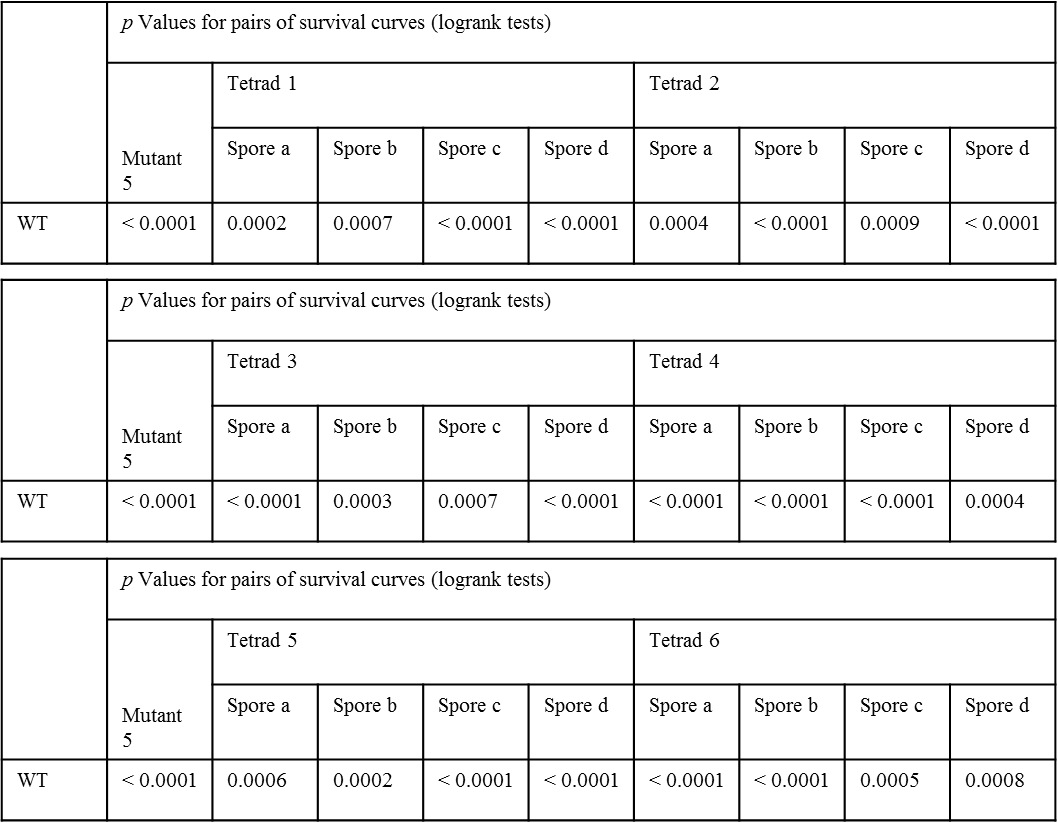


**Supplementary Figure 6.** **Chronologically aging cells of all four ascospores within each of the six randomly chosen tetrads that originated from the WT 🞨 5 diploid exhibit an extended CLS characteristic of the parental mutant strain 5 and live significantly longer than cells of the parental WT strain.** The parental WT strain BY4742, the long-lived parental mutant strain 5 (in the BY4742 genetic background) and the four ascospores recovered from each of the six randomly chosen tetrads were cultured in YP medium without LCA initially containing 0.2% glucose. Survival curves shown in Supplemental Figure 5 were used to calculate *p* values for various pairs of survival curves of different yeast strains. The survival curve for the WT strain was considered statistically different from the survival curve for the mutant strain 5 or from the survival curve for the ascospore if the *p* value was less than 0.05. The *p* values for comparing pairs of survival curves using the logrank test were calculated as described in Materials and Methods.


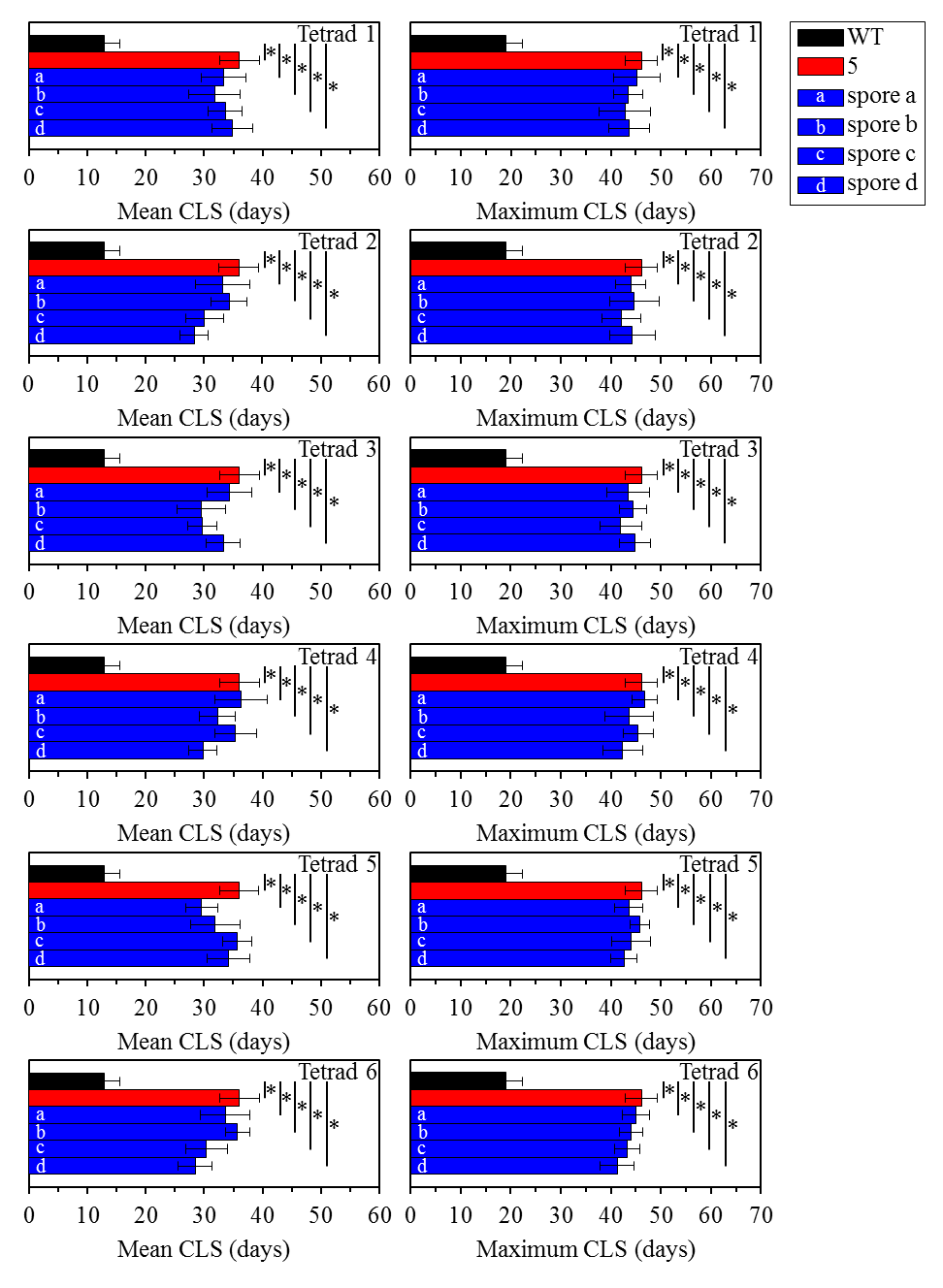


**Supplementary Figure 7.** **Chronologically aging cells of all four ascospores within each of the six randomly chosen tetrads that originated from the WT 🞨 5 diploid exhibit an extended CLS characteristic of the parental mutant strain 5 and live significantly longer than cells of the parental WT strain.** The parental WT strain BY4742, the long-lived parental mutant strain 5 (in the BY4742 genetic background) and the four ascospores recovered from each of the six randomly chosen tetrads were cultured in YP medium without LCA initially containing 0.2% glucose. Survival curves shown in Supplemental Figure 5 were used to calculate the mean and maximum CLS for different yeast strains. Data are presented as means ± SEM (n = 6 for the parental WT strain; n = 2 for the parental mutant strain and for ascospores a, b, c and d; *p < 0.05).


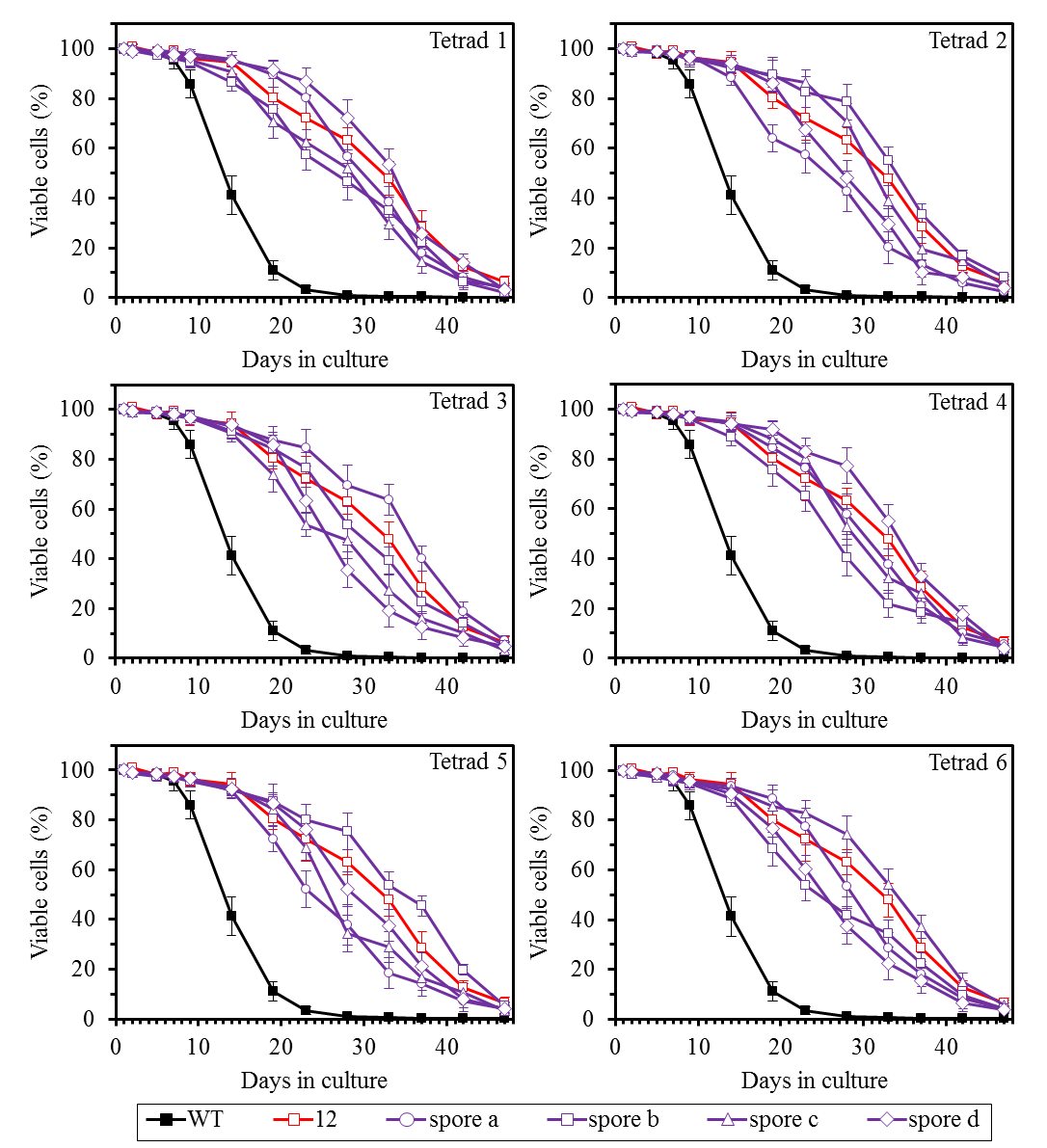


**Supplementary Figure 8.** **Chronologically aging cells of all four ascospores within each of the six randomly chosen tetrads that originated from the WT 🞨 12 diploid exhibit an extended CLS characteristic of the parental mutant strain 12 and live significantly longer than cells of the parental WT strain.** The parental WT strain BY4742, the long-lived parental mutant strain 12 (in the BY4742 genetic background) and the four ascospores recovered from each of the six randomly chosen tetrads were cultured in YP medium without LCA initially containing 0.2% glucose. Survival curves of chronologically aging cells are shown. Data are presented as means ± SEM (n = 6 for the parental WT strain; n = 2 for the parental mutant strain and for ascospores a, b, c and d).


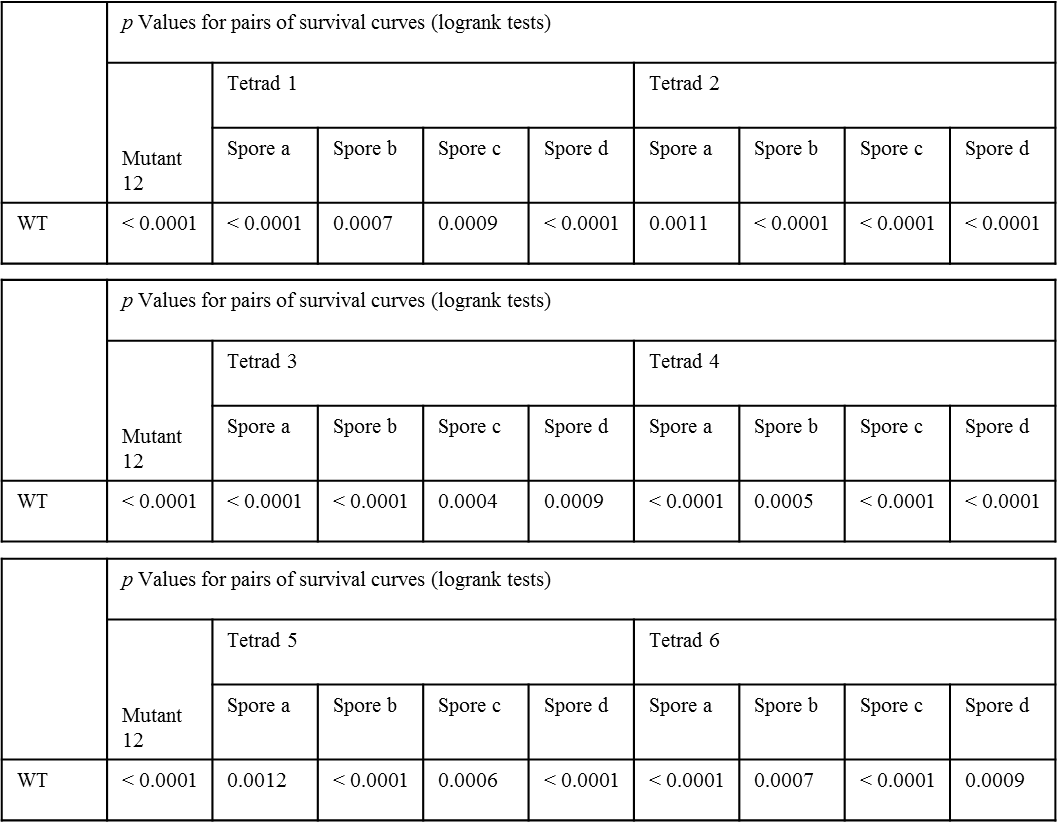


**Supplementary Figure 9.** **Chronologically aging cells of all four ascospores within each of the six randomly chosen tetrads that originated from the WT 🞨 12 diploid exhibit an extended CLS characteristic of the parental mutant strain 12 and live significantly longer than cells of the parental WT strain.** The parental WT strain BY4742, the long-lived parental mutant strain 12 (in the BY4742 genetic background) and the four ascospores recovered from each of the six randomly chosen tetrads were cultured in YP medium without LCA initially containing 0.2% glucose. Survival curves shown in Supplemental Figure 8 were used to calculate *p* values for various pairs of survival curves of different yeast strains. The survival curve for the WT strain was considered statistically different from the survival curve for the mutant strain 12 or from the survival curve for the ascospore if the *p* value was less than 0.05. The *p* values for comparing pairs of survival curves using the logrank test were calculated as described in Materials and Methods.


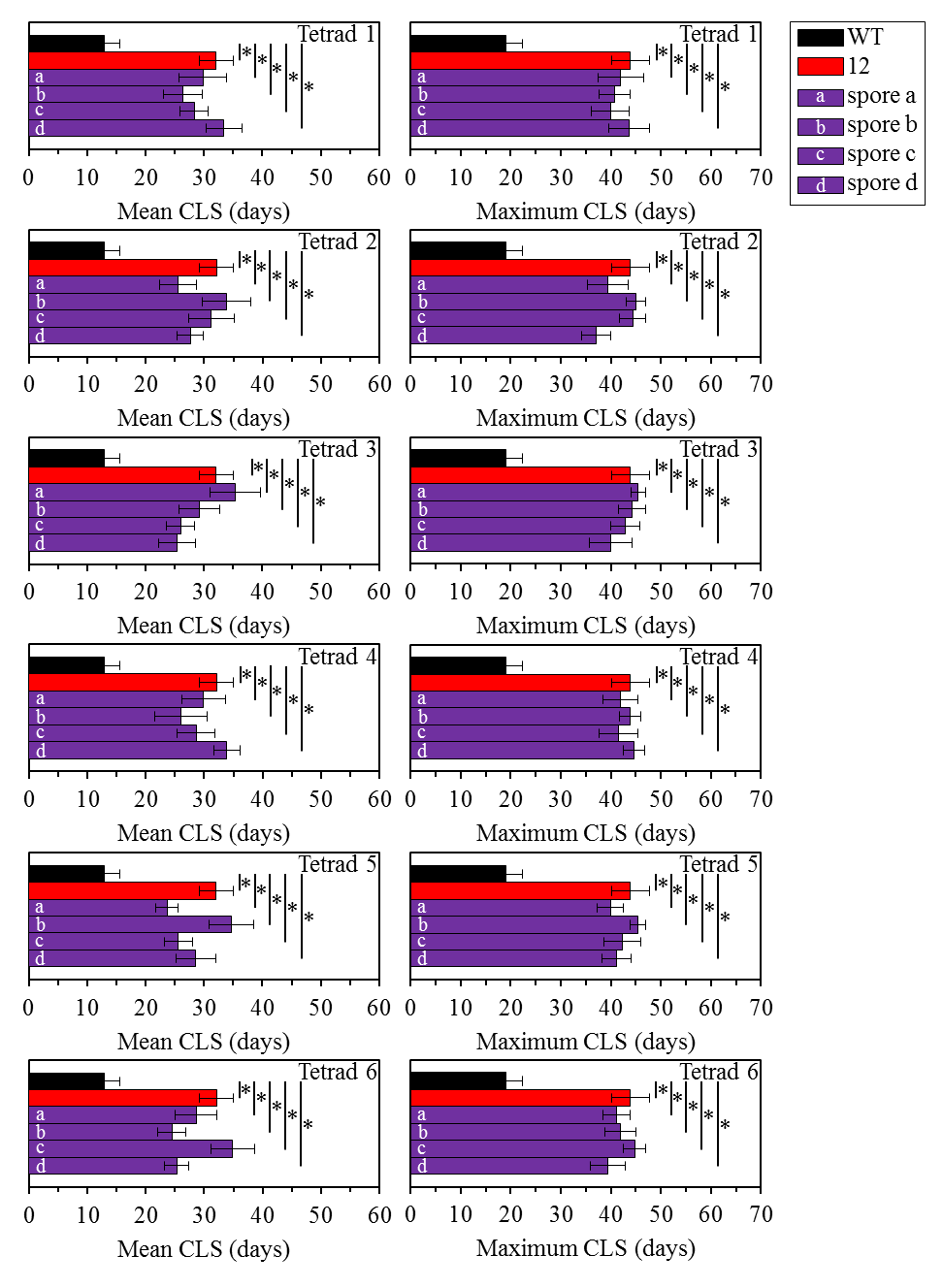


**Supplementary Figure 10.** **Chronologically aging cells of all four ascospores within each of the six randomly chosen tetrads that originated from the WT 🞨 12 diploid exhibit an extended CLS characteristic of the parental mutant strain 12 and live significantly longer than cells of the parental WT strain.** The parental WT strain BY4742, the long-lived parental mutant strain 12 (in the BY4742 genetic background) and the four ascospores recovered from each of the six randomly chosen tetrads were cultured in YP medium without LCA initially containing 0.2% glucose. Survival curves shown in Supplemental Figure 6 were used to calculate the mean and maximum CLS for different yeast strains. Data are presented as means ± SEM (n = 6 for the parental WT strain; n = 2 for the parental mutant strain and for ascospores a, b, c and d; *p < 0.05).
